# Retraction: LIFR-AS1 modulates Sufu to inhibit cell proliferation and migration by miR-197-3p in breast cancer

**DOI:** 10.1042/BSR-2018-0551_RET

**Published:** 2024-11-15

**Keywords:** breast cancer, cell migration, LIFR-AS1, miR-197-3p, Sufu

This article “LIFR-AS1 modulates Sufu to inhibit cell proliferation and migration by miR-197-3p in breast cancer” DOI: 10.1042/BSR20180551 is being retracted from *Bioscience Reports* at the request of the Editor-in-Chief and the Editorial Board. This follows receipt of a notification from a reader, alerting the Editorial Board to samples in Figure 7A which seems to appear in Figure 8a from Jiang et al, 2019 (DOI: 10.1186/s12935-019-0872-4). Additionally, parts of Figure 3A MCF7 0H Control panel seem to appear in Figure 3A MCF7 0H LIFR-AS1 panel with modification.

The Editorial Office was previously contacted by the authors requesting to retract the article, as they had not studied the work completely and new results were discovered. The authors have since been contacted with regards to the concerns raised and the retraction, but have not responded to the journal's queries. Given the issues raised, the Editorial Board stand by the decision to retract the article.

